# Tiny Tummies, Big Challenges: A Case Series of Neonatal Gastric Perforations

**DOI:** 10.7759/cureus.58149

**Published:** 2024-04-12

**Authors:** Yasir A Lone, Santosh K Singh, Aisha Naaz, Chinmay Chetan, Shalvika V Kashyap

**Affiliations:** 1 Pediatric Surgery, Himalayan Institute of Medical Sciences, Swami Rama Himalayan University, Dehradun, IND; 2 Neonatology, Himalayan Institute of Medical Sciences, Swami Rama Himalayan University, Dehradun, IND; 3 General Surgery, Himalayan Institute of Medical Sciences, Swami Rama Himalayan University, Dehradun, IND

**Keywords:** bowel perforation, peritonitis, gastric perforation, neonatal bowel perforation, neonatal gastric perforation

## Abstract

The main aim of this article is to highlight the clinical features indicating gastric perforation in neonates so that prompt surgery can provide a good outcome for an otherwise fatal condition.

Data was collected retrospectively from all neonates who presented to our tertiary care institute with subsequent diagnosis of gastric perforation from January 2020 to December 2023 (three years). Simple statistical analysis involving sums, means, averages, and percentages was used. Five neonates were operated over a period of three years with a diagnosis of gastric perforation. Two of them were spontaneous. Of the remaining three, each one was associated with malrotation, prematurity, and COVID-19. All five cases could be diagnosed with the finding of free gas in the peritoneum on the abdominal radiograph. Overall mortality was 60% (three of five neonates).

Neonatal gastric perforation typically occurs in the first week of life, specifically within the second to seventh day. Symptom onset is usually sudden, with abdominal distension as the first sign, with acidic contents causing severe peritonitis and rapid progression to sepsis and shock. Early diagnosis with subsequent timely resuscitation and surgical repair is crucial to good outcomes. Massive pneumoperitoneum on abdominal radiographs with typical signs in a neonate should raise suspicion of gastric perforation, especially in the first week of life.

## Introduction

Approximately 7% of all gastrointestinal perforations in newborns are in the stomach [1-3]. The reported incidence ranges from 1:2900 to 1:5000 live births [1,4]. Despite its rarity, it should be kept in mind as a differential diagnosis for neonatal gastrointestinal perforation since early diagnosis and intervention improve outcomes. More than 300 cases have been reported since Siebold described the first case in 1825. It was not until 1950 that Legar et al. reported the first successful surgery [2]. Spontaneous gastric perforation is the most common form of non-obstructive perforation of the gastrointestinal tract in newborns [1]. Any mechanical or functional obstruction that leads to secondary perforations, like any distal atresia or web, meconium ileus, malrotation, and so on, must be ruled out [4-10]. In addition, iatrogenic or traumatic causes like aggressive resuscitation or over-enthusiastic nasogastric tube manipulation have to be excluded before the perforation can be considered spontaneous or idiopathic. Gastric perforations are known to be both more common and more severe in preterm neonates [1-3]. Literature on this rare condition in neonates remains dominated by case reports and series with varying speculation on its etiology. Given the low incidence, it remains controversial and incompletely understood. We present the features of gastric perforation in neonates in our experience (five cases within the last three years).

## Case presentation

All neonates presenting to our neonatal and pediatric emergency with subsequent diagnosis of gastric perforation from 1st January 2020 to 31st December 2023 (three years) were included in the study. The departments of pediatric surgery and neonatology at our tertiary care hospital were involved. Five cases met the inclusion criteria.

Case 1

**Figure 1:**
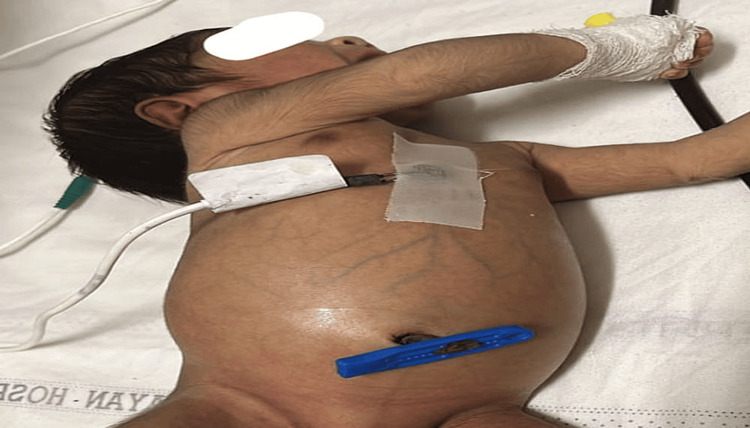
Abdominal distension in case 1

A four-day-old baby boy was brought to our pediatric emergency with a single-day history of excessive crying and abdominal distention (Figure 1).

**Figure 2:**
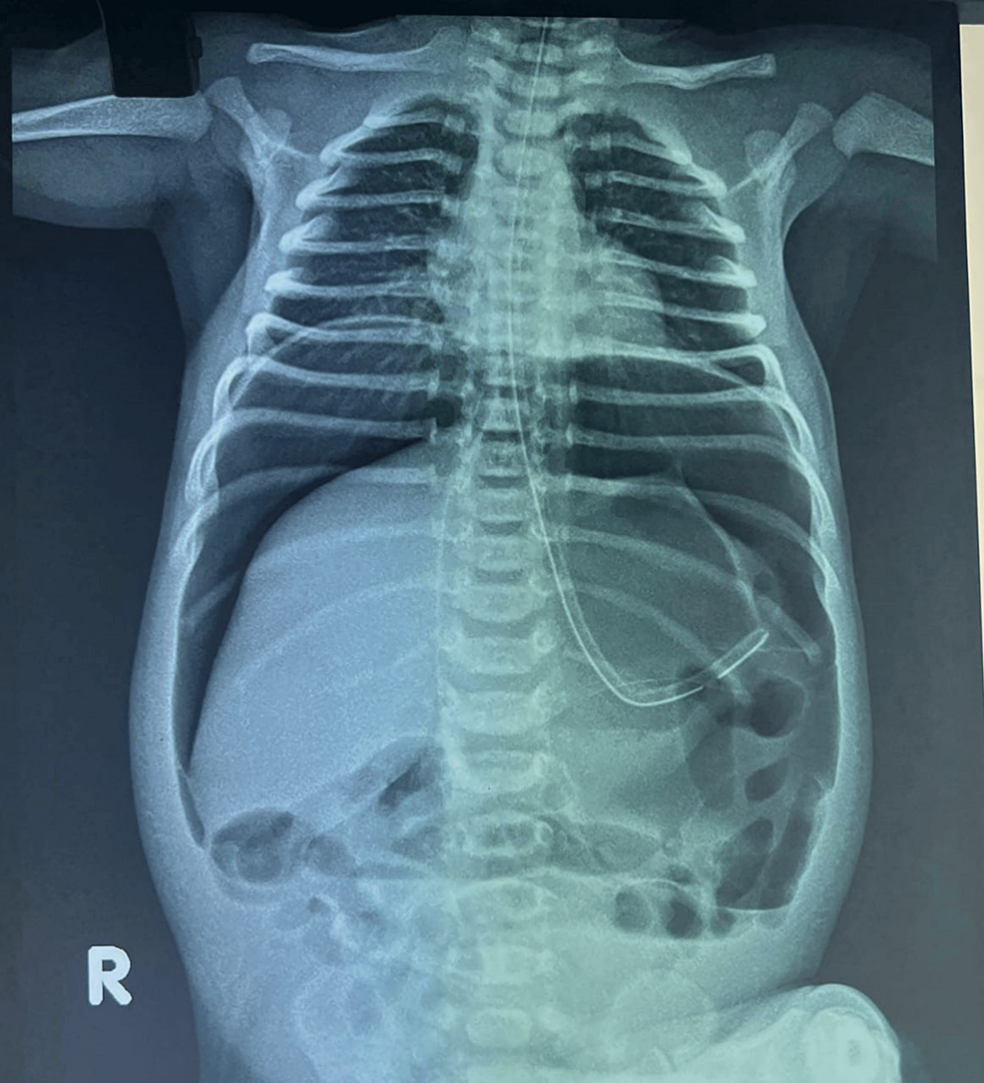
Erect abdominal X-ray of case 1 The image shows huge free gas under both domes of the diaphragm, known as the “continuous diaphragm sign,” and also shows the “falciform ligament sign” and “football sign.”

It was a full-term (37 weeks) normal vaginal delivery at home with a birth weight of 2.7 kg, and the baby cried well after birth, passed meconium within 24 hours of life, and was tolerating feeds well for the initial three days. The only probable concern was a history of being fed cow’s milk once on day one of life. The erect abdominal X-ray revealed huge free gas under both hemidiaphragms (Figure 2).

**Figure 3:**
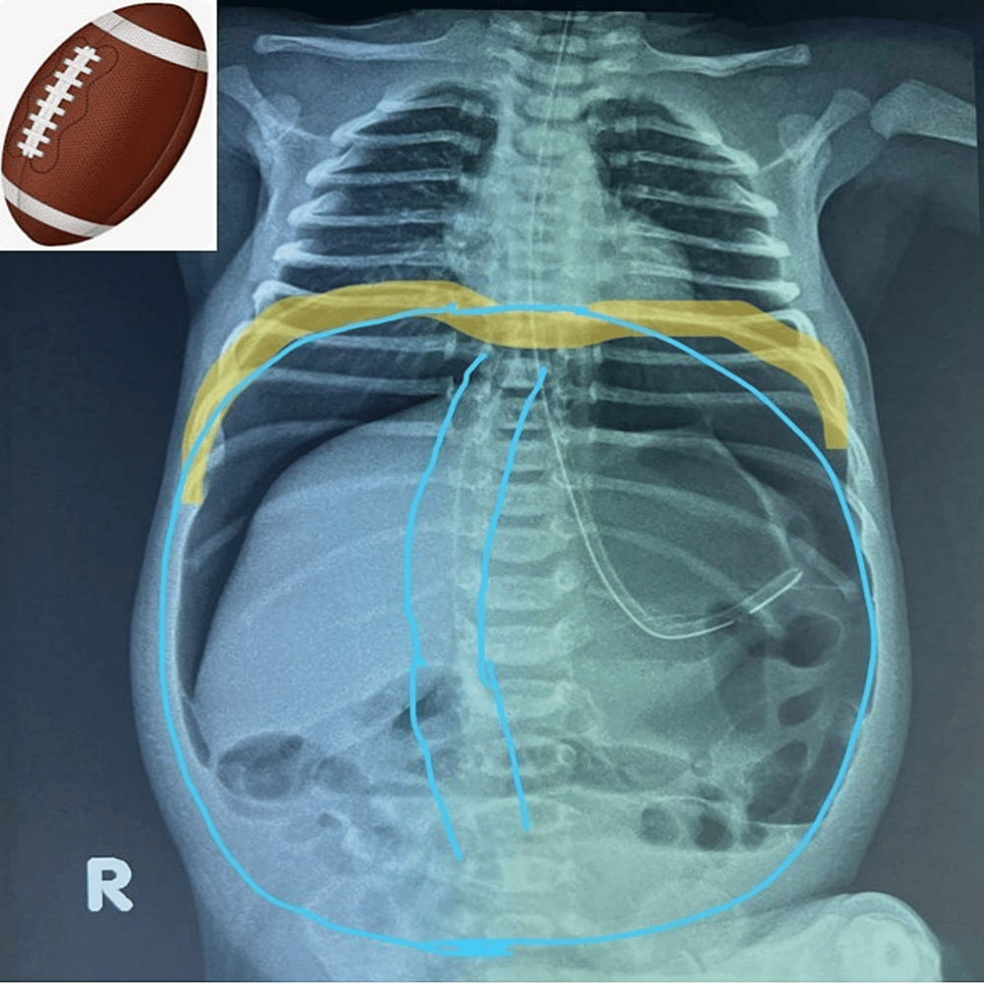
Erect abdominal X-ray of case 1 demonstrating “continuous diaphragm sign” and “football sign”

The “continuous diaphragm sign” and “football sign” were both prominent on the X-ray shown in Figure 3.

After adequate resuscitation and essential initial investigations, the baby was taken up for urgent abdominal exploration.

**Figure 4:**
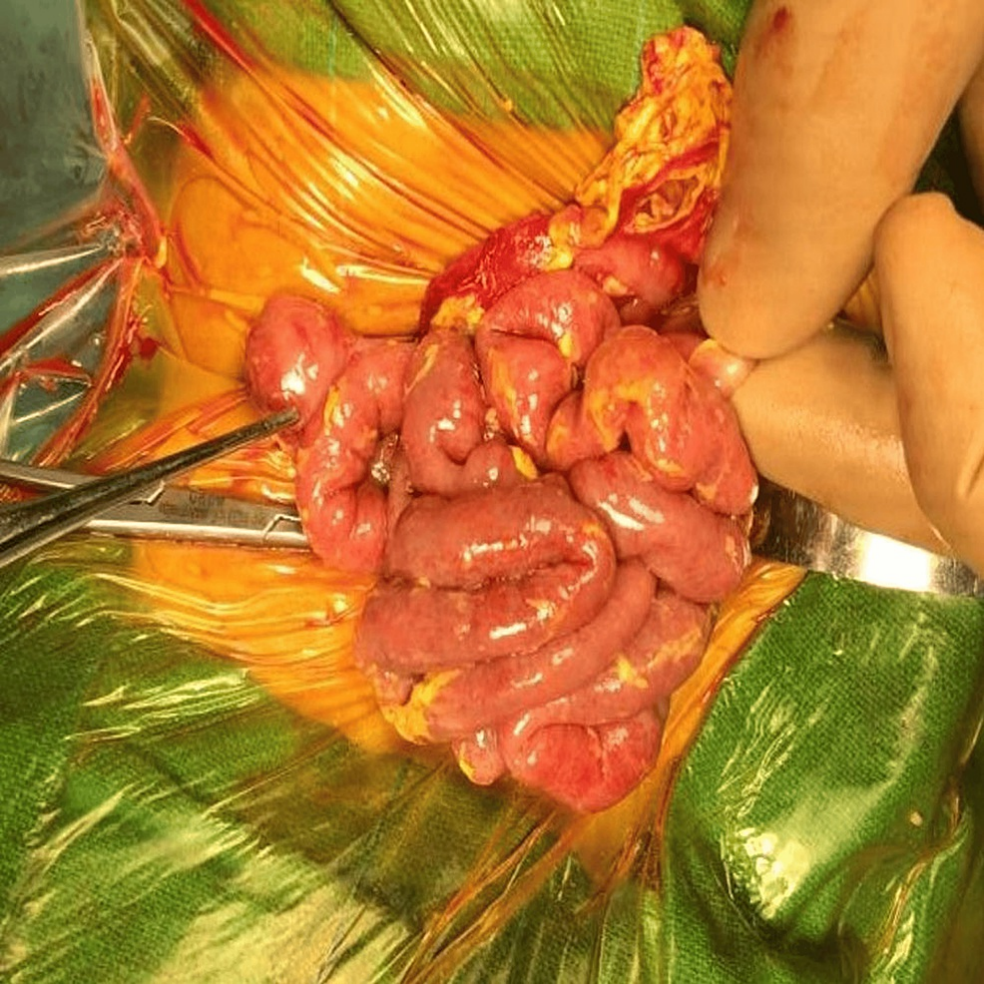
Intraoperative picture of case 1

The peritoneal cavity was filled with purulent fluid, but the entire small and large bowel traced from the duodenojejunal flexure to the rectosigmoid junction near pelvic peritoneal reflection was intact (Figure 4).

**Figure 5:**
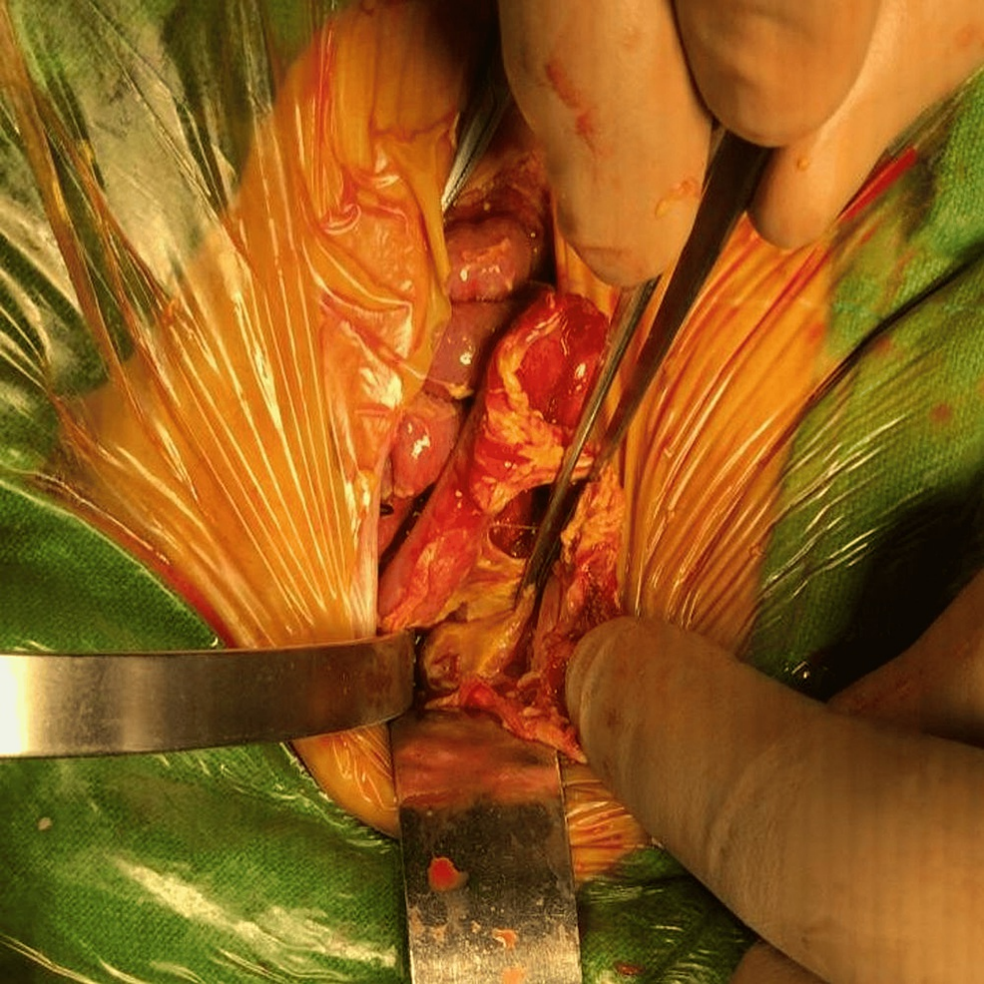
Intraoperative picture of case 1 showing gastric perforation

The bulge from the lesser sac guided us to the posterior gastric wall, which acts as the site of a small 5 mm × 5 mm perforation close to mid greater curve (Figure 5).

After freshening the edges and ensuring a good histopathological specimen, it was repaired primarily with 4-0 polyglactin-interrupted sutures and a glove drain kept near it before closure.

Broad-spectrum antibiotic coverage was continued postoperatively with adequate gram-positive, gram-negative, and anaerobic cover. The peritoneal pus sent for culture was sterile. His serum creatinine of 2.18 normalized after adequate fluid resuscitation. However, the sepsis involved the lungs, and the baby had a prolonged postoperative ventilator requirement of 10 days. The baby was started on minimal orogastric feeds on postoperative day five (POD-5) and gradually reached full feeds. We were able to extubate the baby on POD-10. The wound healed well. The baby was discharged on POD-20 once we were able to demonstrate adequate weight gain and the parents were comfortable taking care of the baby. He was feeding well, achieving developmental milestones, and gaining weight well on the last follow-up about two years later.

Case 2

**Figure 6:**
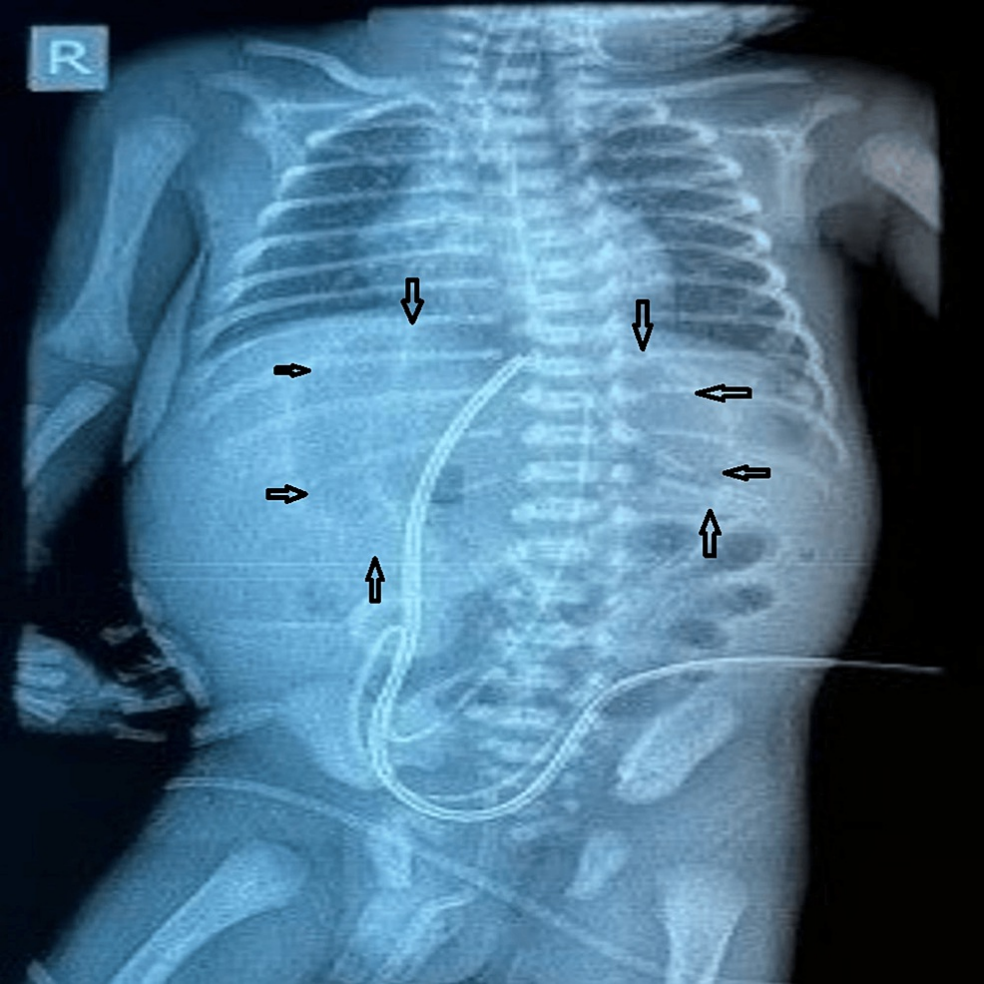
Abdominal radiograph of case 2 Clear demonstration of the “football sign” in case 2. Arrows outline the “football” silhouette.

A 1.4 kg preterm baby boy (30 weeks period of gestation (POG)) was delivered via lower segment cesarean section (LSCS) done for oligohydramnios with premature rupture of membranes. The baby was otherwise active except for some mild respiratory distress, for which some positive airway pressure support was provided. Orogastric (OG) tube feeding commenced on the day of birth, and the baby tolerated it well for the initial six days. He developed abdominal distension on day seven of life and rapidly worsened with features of septic shock. The baby was started on inotropic support, broad-spectrum antibiotics, and appropriate intravenous (IV) fluid resuscitation. Abdominal distension increased subsequently, and abdominal radiography revealed free gas with a “football sign” in the supine X-ray (Figure 6).

In view of rapid worsening and severe distension, an abdominal glove drain was inserted, which drained serohemorrhagic fluid. After reasonably adequate IV fluid resuscitation and stabilization for 48 hours, the baby was taken up for abdominal exploration.

**Figure 7:**
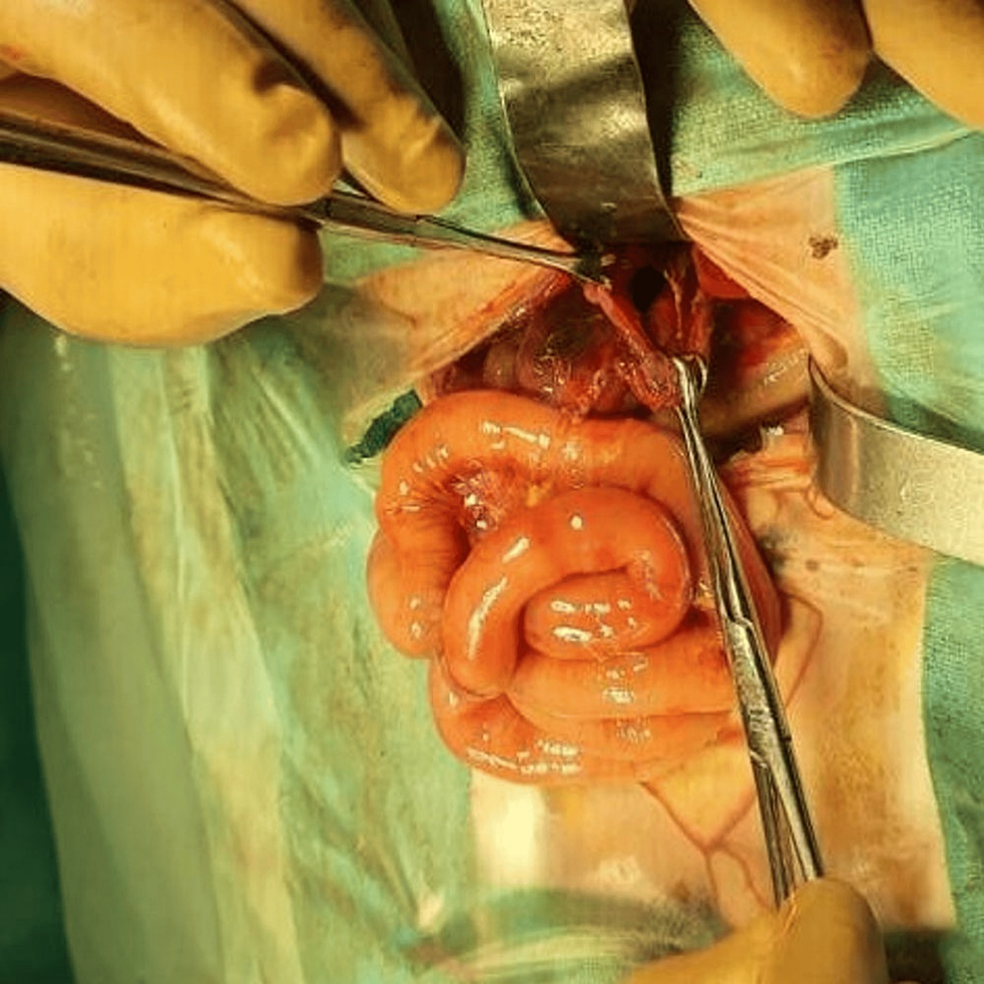
Intraoperative picture of case 2

Intraoperatively, there was a large perforation on the posterior wall of the fundus with adjacent necrosis (Figure 7).

Necrotic margins were excised and repaired in two layers with silk 4-0. Postoperatively, the baby seemed to be recovering well initially. He passed stools on POD-3, and the OG aspirates gradually decreased. After the contrast study on POD-6 revealed no contrast extravasation from the stomach, he tolerated OG feeds well from POD-7. He gradually reached full OG feeds, and we were able to extubate on POD-11. After a few days, he started worsening again and developed mixed respiratory and metabolic acidosis. Inotropes were added, antibiotics were upgraded, and appropriate IV fluid resuscitation was started. He had to be reintubated on POD-16, and his clinical course continued to worsen. He also developed clay-colored stools, and relevant investigations were suggestive of cholestasis. Unfortunately, we lost the baby to sepsis by POD-30 before we could further investigate for cholestasis.

Case 3

**Figure 8:**
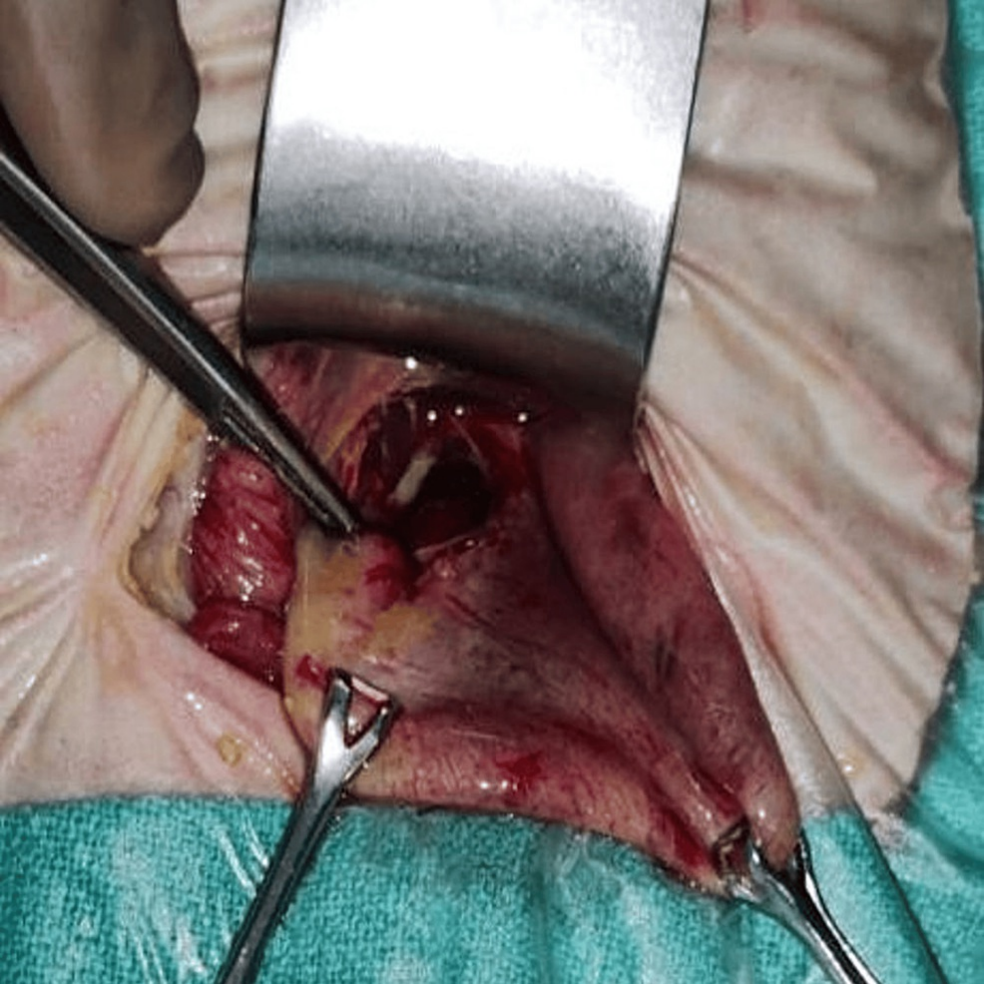
Intraoperative picture of case 3 Intraoperatively, in case 3, a 2 × 3 cm gastric perforation was found along the lesser curve on the anterior wall of the stomach.

A full-term (39 weeks) 2.38 kg baby girl was uneventfully delivered by LSCS (for breech). She passed urine and meconium normally within 24 hours of birth and breastfed. On day two of life, she developed fast breathing and fever, for which she was shifted to the neonatal intensive care unit and kept under observation. On day three of life, the baby developed significant abdominal distension, OG tube bleeding, episodes of sudden bradycardia, and desaturation. The baby had to be intubated and put on ventilatory support, and IV fluid resuscitation, inotropes, and antibiotics were upgraded. Abdominal radiography confirmed pneumoperitoneum. A glove drain inserted in the abdomen relieved the distension somewhat and allowed adequate ventilation. The drainage was only serohemorrhagic and not bilious. After appropriate resuscitation and stabilization for about 48 hours, the baby underwent exploratory laparotomy on day five of life. A gastric perforation of about 2 × 3 cm was found along the lesser curve of the anterior wall of the stomach (Figure 8).

It was repaired primarily with 4-0 polyglactin along with a feeding jejunostomy.

Postoperatively, inotropic support was tapered off by POD-7, and a small amount of jejunostomy feeds were started and gradually increased. The baby was extubated by POD-13. Recovery was slow, and we believed that the baby’s COVID IgG-positive status could be a contributing factor, even though COVID-19 RT-PCR was negative. The baby’s mother was COVID-positive. On day 17 of life, the baby again developed abdominal distension, along with high abdominal glove drain output. On POD-21, the baby was taken up for re-exploration. All previous suture sites were healthy, but there was a small jejunal perforation distal to the jejunostomy site. After repair, the baby again had a prolonged ventilatory requirement. In view of the reported hypercoagulable state associated with COVID-19 [10,11], necrosis with vessel thrombosis seen on histopathological examination of the perforation site and skin biopsy, as well as the elevated d-dimer levels, the baby was started on anticoagulants. Ventilatory settings were gradually tapered off, and the baby was extubated on day 21 of life. Small amounts of OG feeds were started and gradually increased. The baby was discharged when she was accepting breastfeeding well, the wounds had healed, and she was gaining weight adequately. On the last follow-up about two years later, the baby was feeding well and showing satisfactory progress.

Case 4

**Figure 9:**
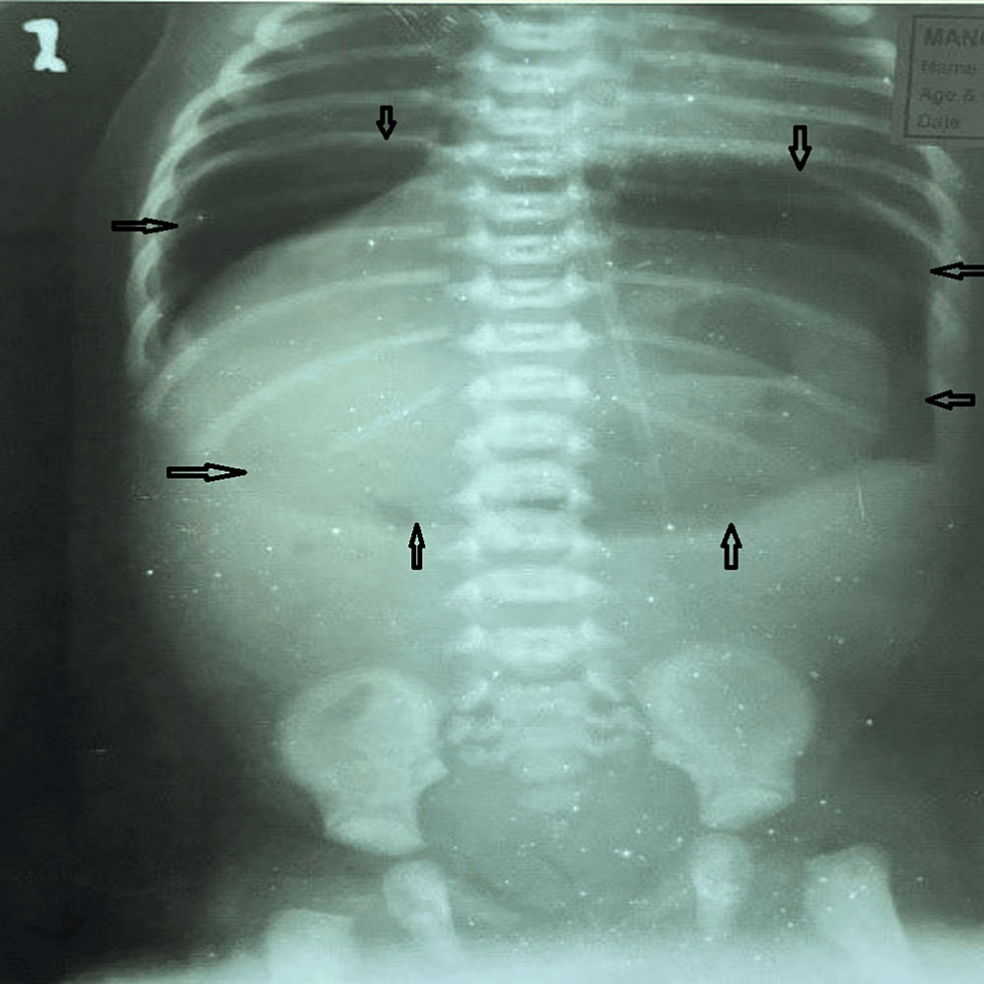
Erect abdominal X-ray of case 4 Erect X-ray in case 4 revealed the “continuous diaphragm sign” and “football sign.” Arrows outline the “football” silhouette.

Eleven days old full-term (38 weeks) baby boy born by normal vaginal delivery (birth weight, 3.45 kg) presented to our pediatric emergency with parents complaining about abdominal distension and shortness of breath for the last three days. An X-ray of the abdomen was done, which confirmed a huge pneumoperitoneum along with a “continuous diaphragm sign” and “football sign” (Figure 9).

**Figure 10:**
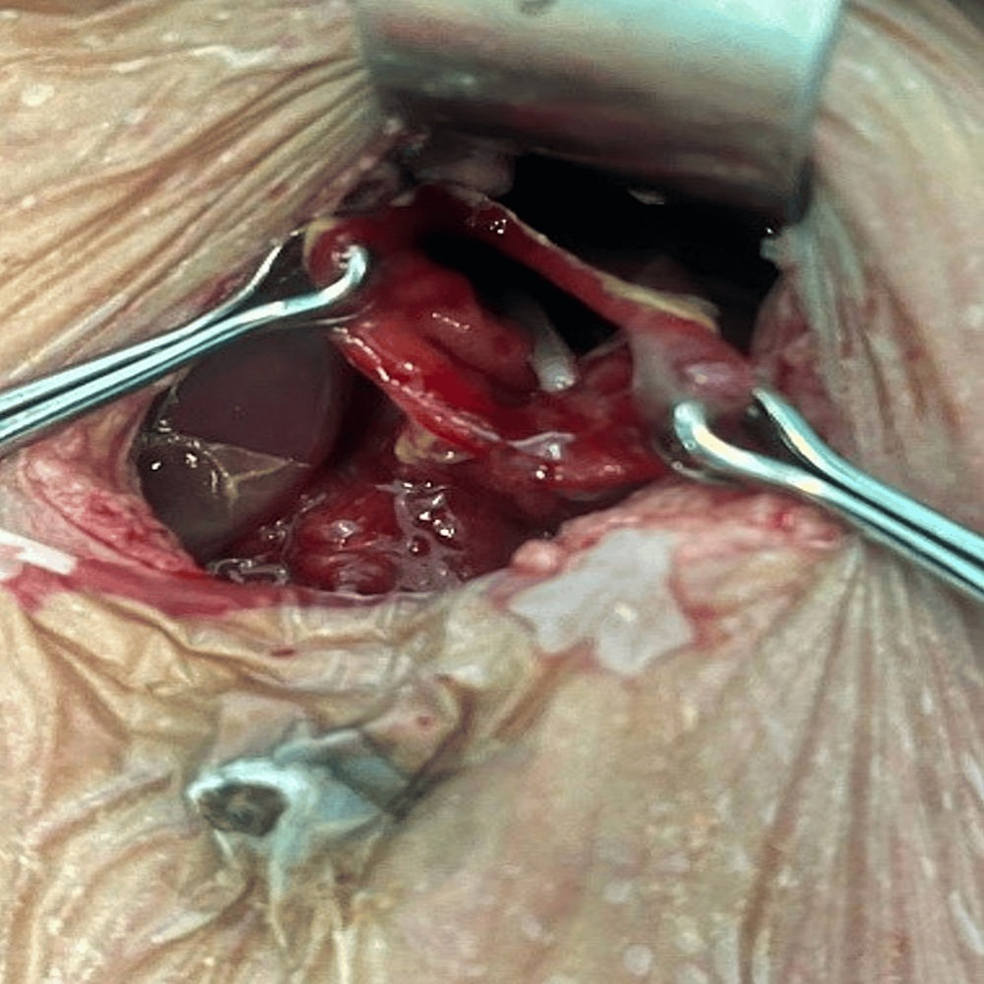
Intraoperative picture of case 4 Intraoperatively, in case 4, there was a 4 × 3 cm gastric perforation on the anterior wall near the cardia.

The patient was hemodynamically unstable and unfit for surgery. He was admitted to our neonatal intensive care unit for initial resuscitation, and an abdominal drain was inserted. The baby was eventually taken up for exploratory laparotomy on day 13 of life (after 48 hours). Intraoperatively, there was a large gastric perforation of size roughly 4 × 3 cm on the anterior wall of the stomach extending toward the cardia (Figure 10).

Primary repair of the gastric perforation was done with drain placement in the subhepatic region. Postoperatively, the baby was on ventilatory support and was intermittently weaned but could never completely recover. By POD-4, the baby had developed severe sepsis and multi-organ dysfunction and eventually expired.

Case 5

**Figure 11:**
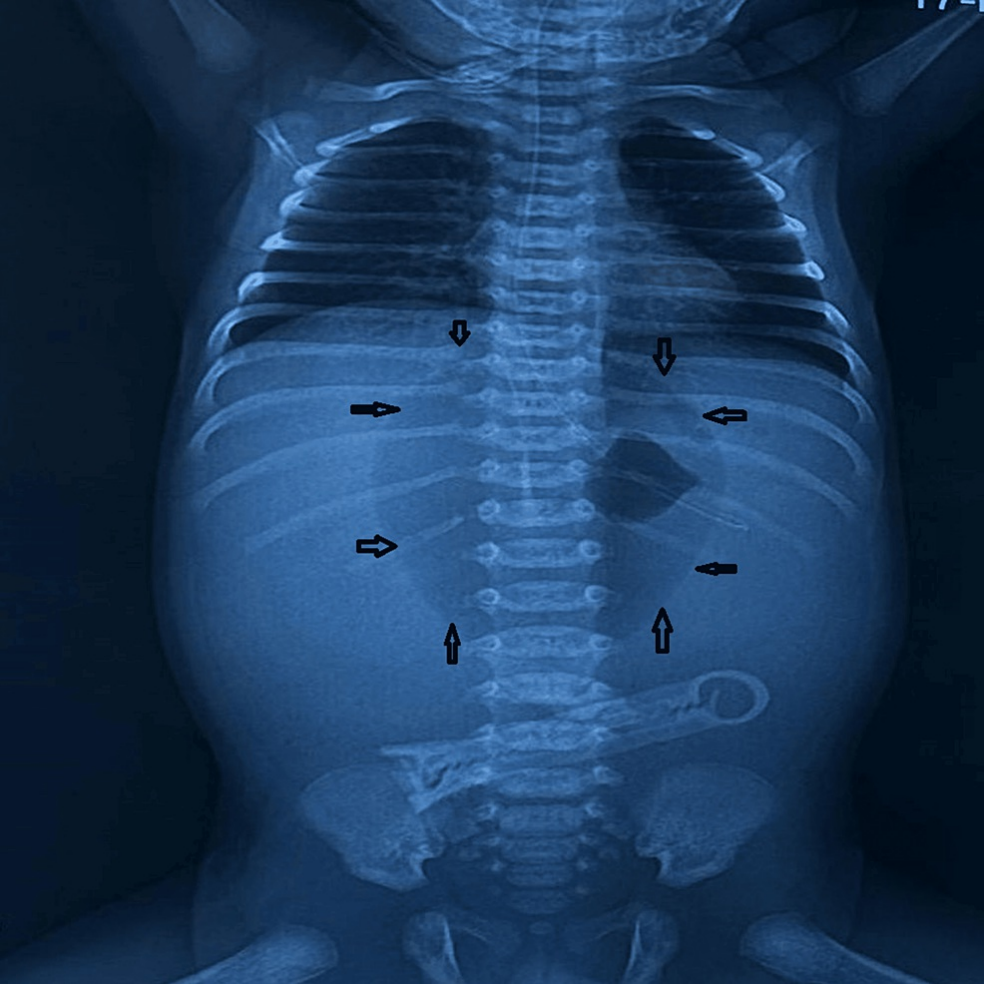
Abdominal radiograph of case 5 An X-ray of the abdomen in case 5 revealed a “football sign.” Arrows outline the “football” silhouette.

A full-term baby boy (birth weight, 2.5 kg) born by LSCS presented on day six of life to our pediatric emergency with complaints of abdominal distension and multiple episodes of vomiting for the last three days. An X-ray of the abdomen was done, which confirmed pneumoperitoneum and “football sign” on the supine X-ray abdomen (Figure 11).

**Figure 12:**
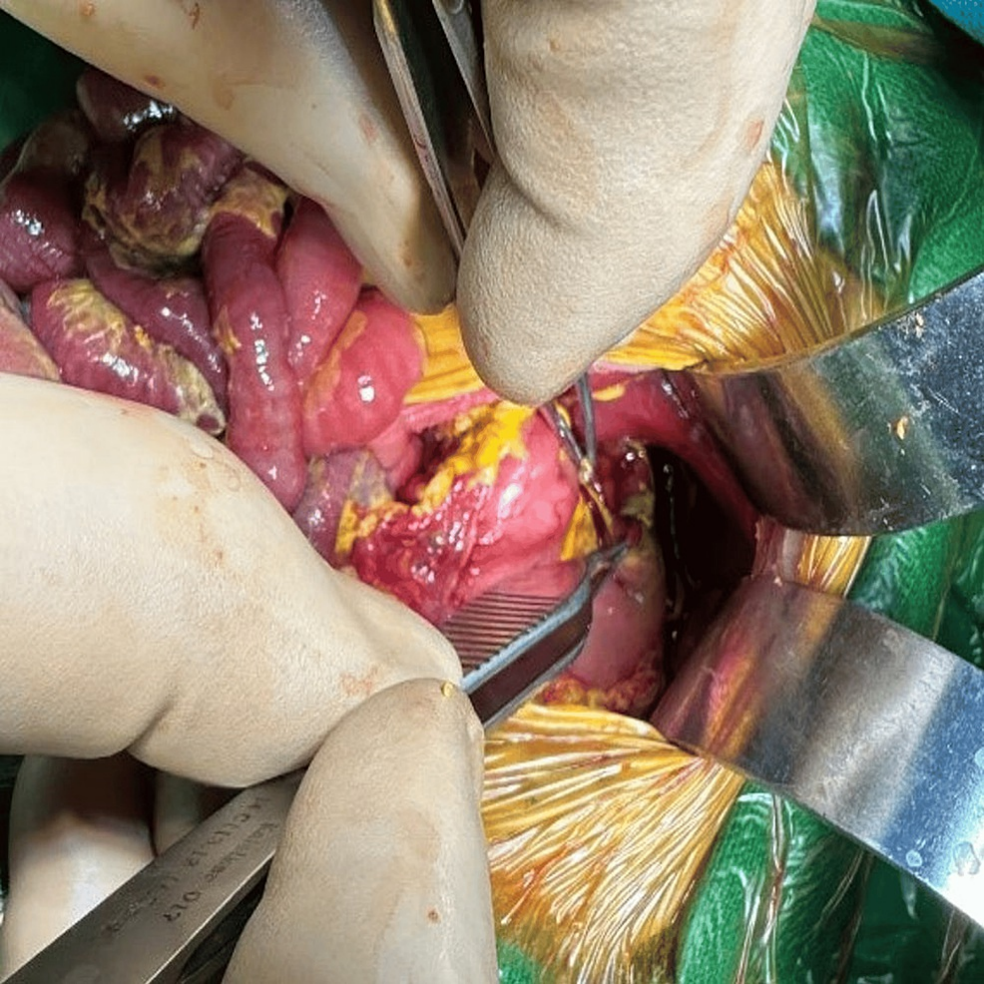
Intraoperative picture of case 5 A gastric perforation in case 5 on the anterior wall near the gastroesophageal junction

On exploratory laparotomy, an anterior gastric wall perforation of size roughly 2 × 3 cm was found along the greater curve of the fundus near the gastroesophageal junction (Figure 12).

**Figure 13:**
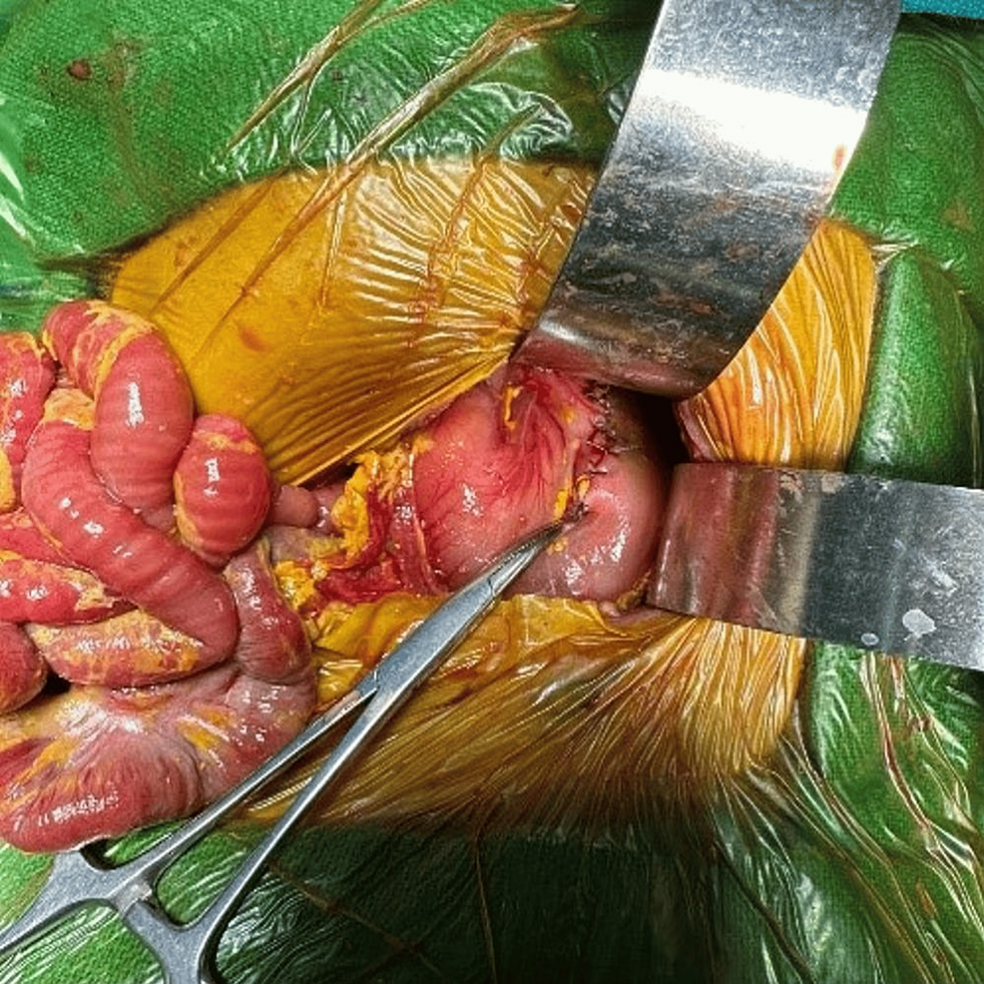
Gastric perforation in case 5 after repair

 It was primarily repaired with 4-0 polyglactin (Figure 13), and an abdominal drain was kept in the subhepatic space.

**Figure 14:**
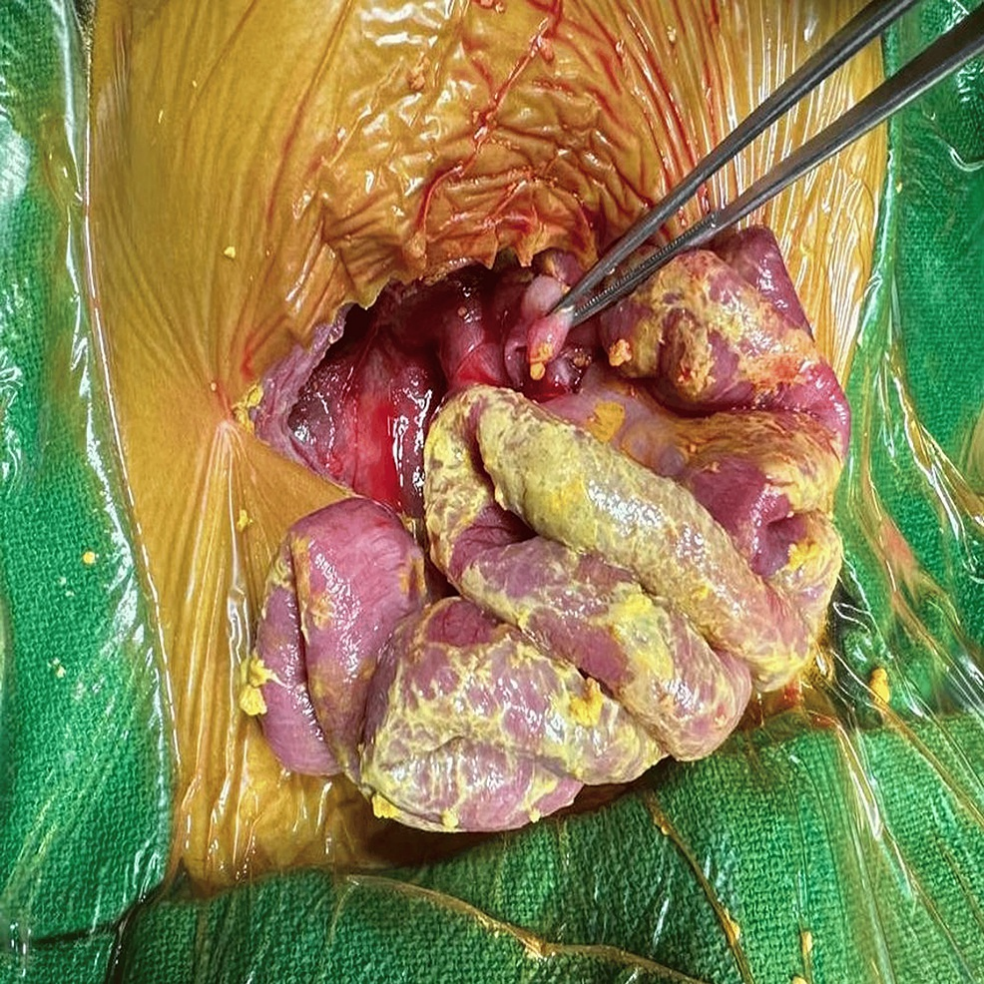
Malrotation in case 5 In case 5, the cecum was pulled up (appendix as an indicator), and there were Ladd's bands across the second part of the duodenum intraoperatively. intraoperatively.

Ladd’s bands were also found along with malrotation (Figure 14).

**Figure 15:**
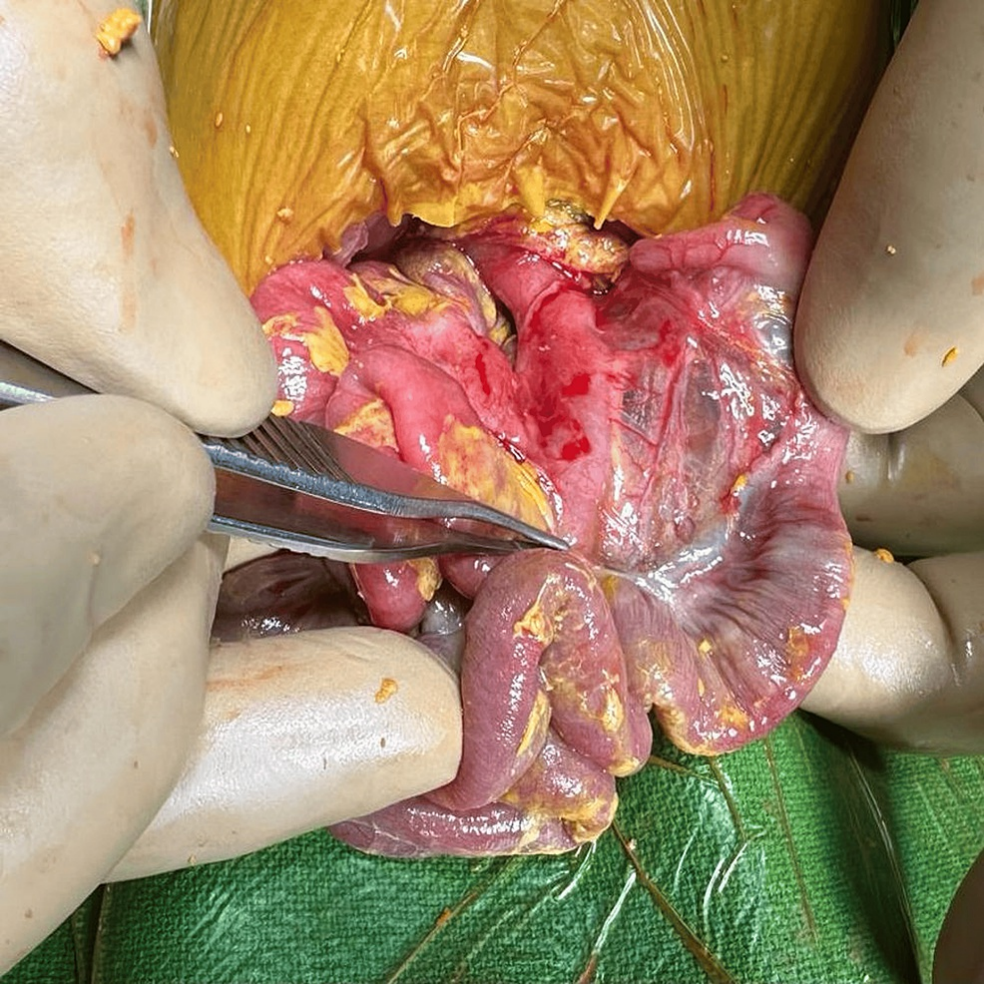
Ladd’s procedure done in case 5 Ladd’s procedure in case 5 involved straightening the duodenum and widening the mesentery.

Ladd’s procedure was done to correct the malrotation (Figure 15).

The baby showed initial transient improvement with a soft abdomen and lowered ventilatory requirements, but he never completely recovered. The sepsis started worsening from POD-6 with increasing inotropic requirement. The baby eventually expired on POD-10.

A summary of all the five cases is provided in Table 1.

**Table 1:** Table summarizing the chief clinical features of the 5 cases in the case series

S. no.	Gestational age; age at presentation	Sex	Chief complaint	Operative findings	Outcome
1	37 weeks; day 4	M	Abdominal distension	0.5 x 0.5 cm perforation on the posterior wall, close to the greater curve	Doing well on the last follow-up about 2 years later
2	30 weeks; day 7	M	Abdominal distension	Large perforation on the posterior wall along the fundus	Mortality
3	39 weeks; day 2	F	Abdominal distension	2 x 1 cm perforation on the anterior wall along the lesser curve	Doing well on the last follow-up about 2 years later
4	38 weeks; day 11	M	Abdominal distension, respiratory distress	4 x 3 cm perforation on the anterior wall extending to the cardia	Mortality
5	37 weeks; day 6	M	Abdominal distension, multiple episodes of vomiting	Malrotation; 2 x 3 cm perforation on the anterior wall	Mortality

## Discussion

A trio of mechanisms have been put forward as possible causes for gastric perforation in neonates: traumatic, ischemic, and spontaneous. Most are because of iatrogenic trauma by over-enthusiastic NG or OG tube manipulation. Appearing as a tiny hole or a small laceration, the perforation usually happens on the greater curve [2]. 

Ischemic perforations are largely due to prematurity, possibly due to general immaturity of the layers of the stomach and its protective factors. The mechanism of ischemic perforation is tough to describe because they are linked with conditions of profound physiological stress, such as severe prematurity, sepsis, and neonatal asphyxia. Any hypoxic stress is known to cause gastric ischemia by selective shunting of blood away from splanchnic vasculature. Since gastric stress ulcers are known to occur in critically ill infants, these perforations may be a result of transmural necrosis of such ulcers [2]. Another hypothesis proposes congenital anatomical defects in the gastric muscle wall as potentiating factors for perforation, especially in premature neonates. The neonatal gastric circular muscle layer typically has multiple gaps, most commonly in the fundus and along the greater curve. They are more prominent in prematurity [5]. Also, mechanical rupture of the gastric wall secondary to elevated gastric pressure due to incoordination or lack of maturity of the vomiting mechanism in newborns, especially preterm babies, maybe a contributing factor. So, a combination of these factors may be responsible for more extensive necrotic perforations in premature neonates, as in case 2 of our series. 

Spontaneous gastric perforation reports have surfaced from otherwise full-term and fit neonates, usually within the first week of life, specifically between the first two to seven days [2,6]. The baby in case 1 of our series, nevertheless, was full term, otherwise healthy at birth, and presented within the classical first two to seven days of life described. Miller et al. found that gastric acidity in neonates and adults is the same, peaking at 24 hours of age [3]. This decreases over the following nine days, reaching the usual level for children. So, gastric acidity is profoundly higher in the first seven days of life. Occurrence of gastric perforation also peaks at the same time [3]. Additionally, poor nutrition, gastric mucus secretion, and digestion ability in preterm neonates may increase the risk and severity of stomach perforation. Nevertheless, additional research is necessary to explain the early appearance of gastric perforation in preterm babies. 

The real etiology is still not completely understood. Some cases may be secondary to distal obstruction like malrotation, as in case 5 of our series. Though gastric perforations have been described as secondary to iatrogenic trauma and distal obstruction like pyloric web, malrotation, and others [3,8], these secondary causes must be ruled out before we can actually call them “spontaneous,” as in case 1 or case 4 of our series. Gastric perforations have also been reported in association with drugs like steroids and cyclooxygenase inhibitors for ductal closure [3,8,9]. Up to 20% of patients with COVID-19 might have dysgeusia, nausea, vomiting, and diarrhea. However, severe GI complications are rare. Mesenteric ischemia and intestinal perforation, though unusual associations of COVID-19, deserve to be explored in more detail [10,11]. A combination of these and previously mentioned factors may contribute to more extensive disease in case 3 of our series. *Helicobacter pylori* is a bacteria that causes gastritis in humans. Though the infection rate increases with age, higher rates have been reported among young people in the developing countries of the world, where poor sanitation is thought to contribute to an overall high prevalence of *H. pylori* infection. The infection, however, has rarely been reported in infants [12]. 

More than one perforation does occur, but more commonly (85-90%), there is only one linear perforation, several centimeters deep at the greater gastric curve. In case of a punctate perforation, a traumatic factor like the use of nasogastric tubes should be thought of [1,2]. 

Diagnosis is commonly made in the first week of life regardless of its spontaneous character [2,3]. Symptom onset is usually sudden, with abdominal distension as the first sign. It is usually profound and capable of causing respiratory compromise. Vomiting may or may not be present at the time of presentation. Peritonitis due to highly acidic gastric contents may be responsible for rapid progression to sepsis and shock, hence the need for early suspicion, diagnosis, and surgical repair. Gastric perforation should always be kept in mind as one of the most important causes of pneumoperitoneum in the first week of life. Differential diagnoses include necrotizing enterocolitis (NEC), perforation secondary to other causes of intestinal obstruction in neonates like bowel atresia, meconium ileus, etc., and spontaneous pneumoperitoneum. 

Another interesting observation in our series, consistent with the findings in the literature, is the massive pneumoperitoneum on the erect radiograph of a neonate, especially in the absence of stomach gas bubble, bowel air-fluid levels, and pneumatosis intestinalis, suggesting perforation of the stomach rather than any other part of the neonatal gastrointestinal tract. It is usually abundant, and an accentuating falciform ligament (“falciform ligament sign”, Figures 1b, 1c) with air on both sides of the falciform ligament is considered a useful finding in perforation of the stomach or duodenal bulb [13]. If free air is found in the lesser sac (“lesser sac sign”), then the likely site of perforation is the posterior wall of the stomach or near the gastroesophageal junction, fundus, or duodenum [12,13]. Gastric perforation is rarely correlated with air trapped in the mesenteric or sigmoid recess, in contrast to perforation of the colon or small bowel [13]. Massive pneumoperitoneum can present as the “continuous diaphragm sign,” which simply means free gas under both hemidiaphragms, or as a “football sign” resembling the oval translucency of an American football (Figures 1b, 1c) [13,14]. Cases 1 and 4 in our series clearly demonstrate these signs (Figures 1b, 1c, 2a). Some authors have described the “football sign” only for supine X-rays, as in cases 2 and 5 of our series (Figures 1f, 2c), indicating that this term should be used only when seen isolated in the absence of free gas under the diaphragm. Even though the underlying disorders of pneumoperitoneum vary, the football sign is most commonly encountered in infants with gastric perforation. Other causes include acute necrotizing enterocolitis, malrotation with midgut volvulus, Hirschsprung’s disease, meconium ileus, intestinal atresia, and peptic ulcers [14]. It is rarely seen in older children and hardly in adults [13]. The large free gas under pressure probably stretches the thinner and more elastic neonatal abdominal wall, allowing it to acquire this peculiar football contour. Nevertheless, one should be tuned to pick up these early signs since early diagnosis and prompt surgical intervention are crucial to a good recovery. 

Aggressive resuscitation, prompt antibiotic coverage, and intensive monitoring and care become essential for good outcomes. During the insertion of an abdominal glove drain for relieving massive abdominal distension, if the drainage is largely air and serous fluid instead of bilious or feculent, it again points toward the location of perforation being proximal to the second part of the duodenum. After adequate resuscitation, the only treatment is urgent surgical repair. Intraoperatively, the entire gastrointestinal tract should be examined for secondary causes and other perforations. 

The mortality approaches 50%, and therefore, there is a need to reiterate timely diagnosis and surgery [1,2]. Unfavorable prognostic factors include male sex, hyponatremia (serum sodium, <130 mmol/L), and metabolic acidosis (pH, <7.3), as mentioned in the literature. However, recent reviews from the last decade suggest that early diagnosis and subsequent timely resuscitation and surgery lead to favorable outcomes because advancements in neonatal intensive care have improved our ability to correct metabolic and electrolyte abnormalities before they become irreversible. According to their systematic review in 2018, Chen et al. reported mortality rates in the preterm and term subgroups as 72.4% and 33.3%, respectively [3]. Therefore, preterm newborns have a 4.21 times higher risk of mortality than full-term newborns with perforation of the stomach. Furthermore, low birth weight was found to be an independent risk factor for higher mortality, even in full-term neonates [2,3]. This could explain the unfavorable outcome in case 2 of our series.

## Conclusions

Gastric perforation should always be kept in mind as one of the most important causes of pneumoperitoneum in the first week of life. Symptom onset is usually sudden, with abdominal distension as the first sign and acidic contents causing severe peritonitis with rapid progression to sepsis and shock. Early diagnosis with subsequent timely resuscitation and surgical repair is therefore crucial to good outcomes. Massive pneumoperitoneum on erect radiograph of a neonate, especially in the absence of gastric air bubble, bowel air-fluid levels, and pneumatosis intestinalis, should be considered an indicator of perforation of the stomach rather than any other part of the neonatal gastrointestinal tract. One should be tuned to pick up early indicators like “continuous diaphragm sign,” “football sign,” and ‘lesser sac sign,” since prompt surgical intervention is crucial to good outcomes for an otherwise high mortality condition. Preterm newborns have a 4.21 times higher risk of mortality than full-term newborns with perforation of the stomach. Furthermore, low birth weight was found to be an independent risk factor for higher mortality, even in full-term neonates.
